# Ruxolitinib Treatment in a Patient with Primary Myelofibrosis Resistant to Conventional Therapies and Splenectomy: A Case Report

**DOI:** 10.4274/tjh.2013.0338

**Published:** 2015-05-08

**Authors:** Meltem Aylı, Muhit Özcan, Güldane Cengiz Seval

**Affiliations:** 1 Ufuk University Faculty of Medicine, Department of Hematology, Ankara, Turkey; 2 Ankara University Faculty of Medicine, Department of Hematology, Ankara, Turkey

**Keywords:** Primary myelofibrosis, Ruxolitinib, splenectomy

## Abstract

A 67-year-old male patient who was diagnosed with primary myelofibrosis 4 years ago did not respond to conventional therapies. The splenomegaly progressively increased, which caused spleen infarctions and led to the decision to perform a splenectomy procedure. After splenectomy, the patient started treatment with ruxolitinib. In the first month of ruxolitinib treatment, the patient became transfusion-free and all constitutional symptoms disappeared. However, in the sixth month of ruxolitinib treatment, the disease transformed to acute myeloblastic leukemia, and the patient died 1 month later. This is the first case report that shows the effects of ruxolitinib in a splenectomized patient.

## Abstract (tr)

Dört yıl önce primer myelofibrozis tanısı konulan 67 yaşındaki erkek hastaya uygulanan konvansiyonel tedavi yöntemleri ile sonuç alınamadı. Dalak boyutları ileri derecede arttı, hastanın tekrar ayda 4-6 ünite transfüzyon gereksinimi olmaya başladı. Bu dönemde dev boyutlara ulaşan dalakta infarktüs gelişti ve hastaya splenektomi yaptırıldı. Splenektomi sonrası hastaya ruxolitinib başlandı. Ruxolitinib tedavisinin 1. ayından itibaren hasta transfüzyon bağımsız hale geldi, tüm konstitüsyonel semptomlar ortadan kalktı. Ancak ruxolitinib tedavisinin 6. ayında hasta akut myeloblastik lösemiye (AML) transfore oldu. Ve AML tedavisinin 1. ayında hasta kaybedildi. Bu olgu splenektomi yapılmış bir hastada ruxolitinib etkisini gösteren ilk olgudur.

## INTRODUCTION

Primary myelofibrosis (PMF) is a myeloproliferative neoplasm characterized by stem cell-derived clonal myeloproliferation, hypersensitivity to cytokines, reactive bone marrow fibrosis, and extramedullary hematopoiesis. Clinical manifestations are splenomegaly, severe anemia and cytopenias, constitutional symptoms (e.g., hypercatabolic state, fatigue, night sweats, fever), cachexia, bone pain, osteosclerosis, splenic infarct, pruritus, thrombosis, bleeding, leukemic progression, and shortened survival [1]. The pathogenesis of the disease is currently not understood. PMF is a clonal disorder of the hematopoietic stem cells in which the fibrosis is a reactive process involving the interaction of multiple cytokines, such as platelet-derived growth factor (PDGF), transforming growth factor beta 1 (TGF-β1), basic fibroblast growth factor (bFGF), and vascular endothelial growth factor (VEGF). Recent studies have shown mutations that directly or indirectly lead to the deregulated activation of Janus-activated kinase 2 (JAK2). About half of patients with myelofibrosis carry a gain-of-function mutation in the Janus kinase 2 gene (JAK2 V617F) that contributes to the pathophysiology of the disease [2,3]. Conventional medications are largely palliative and rarely provide durable benefits, whereas stem cell transplantation is restricted to a small percentage of patients. These limitations underscore the need to develop more effective disease-targeted therapeutic approaches in patients with myelofibrosis. Appreciation for the activation of JAK2 and the importance of the pathogenesis of myelofibrosis has led to novel therapeutic agents targeting JAKs [4]. 

Ruxolitinib is an orally available and potent selective inhibitor of JAK1 and JAK2, and it is the most advanced JAK1/JAK2 inhibitor in development for the treatment of myeloproliferative neoplasms.

Previous studies showed regression in splenomegaly during ruxolitinib treatment, but there has been no evidence that ruxolitinib has the same effect in splenectomized patients or what the consequences of it are in this patient population. In this case report, we present the results of ruxolitinib treatment in a JAK2 mutation-negative primary myelofibrosis patient who also had a mandatory splenectomy operation. Informed consent was obtained.

## CASE PRESENTATION

A 67-year-old male patient presented to us 4 years ago with a 1-month history of fatigue, night sweats, and abdominal distention. Splenomegaly was observed on physical examination; his spleen was 12 cm below the costal margin. There was no lymphadenopathy. Laboratory findings were as follows: white blood cell (WBC) count was 12,600/mm3, hemoglobin level was 9.0 g/dL with MCV of 86 fL, hematocrit was 26%, erythrocyte count was 3.09x1012/L, platelet count was 450x109/L, and lactate dehydrogenase was 845 IU/L. Peripheral blood smear showed normocytic anemia, tear drop-shaped red blood cells (RBCs) (dacryocytes), and leukoerythroblastosis (nucleated RBCs and granulocyte precursors). The bone marrow aspirate was a dry tap. Bone marrow biopsy revealed an increased number of megakaryocytes and a moderate increase of reticulin fibers. The biopsy results were reported as myelofibrosis. Assays for JAK2 V617F and the Philadelphia chromosome were negative. Chromosomal analysis showed no abnormalities. We investigated the secondary myelofibrosis events, but all of them were negative. These findings showed that the patient had primary myelofibrosis.

The prognostic score of the patient was calculated as intermediate-2 according to the International Prognostic Scoring System. Treatment of myelofibrosis-related anemia was started with androgen (danazol, 600 mg/day). After treatment with danazol for 3 months, it became clear that there was no increase in hemoglobin levels and so danazol treatment was stopped immediately. Treatment of myelofibrosis-related anemia was then started with hydroxyurea but myelosuppression began, and so hydroxyurea treatment was also stopped. In place of hydroxyurea, treatment of myelofibrosis-related anemia was started with interferon-alpha at 3 million IU subcutaneously 3 times/week, but the patient could not tolerate it. In the meantime, he became transfusion-dependent again and needed, on average, 4-6 units of erythrocyte suspension per month. Afterwards, treatment with lenalidomide (25 mg/day each 21 days of 28 days) was started. After this treatment his constitutional symptoms regressed and hemoglobin levels increased, but the splenomegaly never regressed. The patient was followed under lenalidomide treatment for about 18 months. During this period of time, he did not require any transfusions. However, in the 19th month, hemoglobin levels decreased to 6 g/dL and his spleen became enlarged. He gained weight, had night sweats, and became transfusion-dependent again after 4 months. Lenalidomide treatment was stopped and then we applied for compassionate use of ruxolitinib. During the application procedure, the patient’s spleen size increased progressively; because of trouble with spleen infarctions, a splenectomy procedure was performed. Following the first month after splenectomy, ruxolitinib at 10 mg/day was given to the patient, who had a poor prognosis and still needed erythrocyte suspensions. The dosage was carefully raised to 20 mg/day. At the beginning of ruxolitinib treatment, the constitutional symptoms regressed; the patient put on some weight and at the end of the first month he became transfusion-free. However, he did not allow us to perform a control bone marrow biopsy. Therefore, we could not assess the drug’s effect on fibrosis. In the second month of treatment, the weight of the patient returned to its basal level and all of his laboratory values were in the normal reference range. He started working again and had an overseas vacation that summer. He was followed asymptomatically for 6 months, but at the end of the sixth month his disease transformed to acute myeloblastic leukemia. He was rehospitalized and 7+3 chemotherapy was started. The patient died 13 days after rehospitalization because of invasive aspergillosis. 

## DISCUSSION AND REVIEW OF THE LITERATURE

PMF is a rare disease. Patients with PMF usually present with anemia and extramedullary hematopoiesis marked by hepatosplenomegaly that is often accompanied by severe constitutional symptoms. Most patients become RBC transfusion-dependent; progressive splenomegaly is often associated with drug-refractory splenic pain and abdominal discomfort, early satiety, cachexia fatigue, night sweats, and low-grade fever, and some patients develop portal hypertension. Bone marrow examination is essential for the diagnosis of PMF and typically shows granulocytic and megakaryocytic hyperplasia accompanied by variable degrees of bone marrow fibrosis and osteosclerosis [5]. Survival in PMF is affected by the presence of risk factors. In particular, 5 factors have been identified as being independently predictive of poor survival: hemoglobin below 10 g/dL, constitutional symptoms, circulating blast rates of 1% or greater, leukocyte counts of >30x109/L or <4x109/L, and presence of cytogenetic abnormalities. In the absence of these adverse features, median survival exceeds 10 years; in the presence of 1 adverse feature, median survival is shortened to 5 to 10 years; and in the presence of 2 or more adverse features, median survival is less than 5 years [6]. The pathogenesis of PMF is currently not understood. PMF is a clonal disorder of hematopoietic stem cells and fibrosis is a reactive process involving the interaction of multiple cytokines, such as PDGF, TGF-β1, bFGF, and VEGF [3,4,5], produced by CD34+ cells, megakaryocytes, and monocytes.

According to the recommendations of the European LeukemiaNet, the main goals of therapy in PMF are prolongation of survival and, if possible, cure, which is currently only achieved by stem cell transplantation. If prolongation of survival or cure is not possible, symptom-orientated palliation and quality of life are the main goals [7]. Until recently, most treatments provided only palliative care, with no single treatment addressing all of the complications and symptoms of the disorder. Although allogeneic stem cell transplant offers the potential for cure, it is associated with a high mortality rate, even using a protocol of reduced intensity, and thus it is only appropriate for a limited group of patients (e.g., younger, otherwise healthy patients with high-risk myelofibrosis). Splenomegaly is a common sign of PMF that is associated with bothersome symptoms that have a significant negative impact on patients’ quality of life. Until recently, none of the therapies used to treat myelofibrosis were particularly effective in reducing splenomegaly.

Discovery of the JAK2 V617F mutation prompted the development of clinical trials using JAK2 inhibitors; overall, these agents have resulted in meaningful symptomatic improvements and reductions of splenomegaly that were otherwise not achievable with conventional therapy. The suggested mechanism of action of ruxolitinib is attenuation of cytokine signaling via the inhibition of JAK1 and JAK2, resulting in antiproliferative and proapoptotic effects. In the phase III COMFORT-I trial, ruxolitinib demonstrated durable reductions in splenomegaly in patients with myelofibrosis. The proportion of patients that achieved spleen volume reduction of ≥35% from baseline in 24 weeks was 41.9% with ruxolitinib versus 0.7% with the placebo (p<0.0001). In the phase III COMFORT-II trial, reductions in spleen volume of ≥35% were observed in 32% of patients treated with ruxolitinib at week 24 and in 28.5% at week 48 (both p<0.0001). Low toxicity, alleviation of constitutional symptoms, weight gain, and improvement of performance status, exercise capacity, and general physical condition were observed with ruxolitinib treatment [8,9]. In November 2011, the US Food and Drug Administration approved the use of the JAK1- and JAK2-selective inhibitor ruxolitinib for the treatment of patients with intermediate- or high-risk myelofibrosis [10].

Favorable results of ruxolitinib treatment in patients who suffer from PMF are well known, but there is no evidence that shows beneficial effects of the same agent in PMF patients who also had a splenectomy [11]. We observed favorable results with ruxolitinib treatment for 6 months in a PMF-diagnosed patient who had a mandatory splenectomy operation. During this 6-month period, all constitutional symptoms regressed. The patient did not require any erythrocyte suspension transfusions and his clinical status improved. Additionally, no side effects were observed. However, ruxolitinib failed to prevent the disease from transforming into acute myeloblastic leukemia. Ruxolitinib did not demonstrate disease-modifying potential and is not considered a curative therapeutic option.

This case report supports the palliative role of ruxolitinib, which significantly reduced constitutional symptoms in a splenectomized patient. Ruxolitinib improved the patient’s quality of life in a very short time and provided a comfortable life until the return to acute leukemia. This is suggestive of the inability of ruxolitinib to influence the natural history of the disease. The JAK2 V617F mutation may be a key factor, but not necessarily a primary factor, driving the tumorigenesis of PMF. Other molecular events may precede the acquisition of the JAK2 V617F mutation in PMF patients. Novel selective JAK2 inhibitors and other novel agents are the active focus for further clinical investigations.
